# Clinical assessment and FGFR2 mutation analysis in a Chinese family with Crouzon syndrome: A case report

**DOI:** 10.1097/MD.0000000000024991

**Published:** 2021-03-12

**Authors:** Huijun Shi, Jie Yang, Qingmin Guo, Minglian Zhang

**Affiliations:** Department of Ophthalmology, Hebei Eye Hospital, Xingtai, Hebei, China.

**Keywords:** Crouzon syndrome, FGFR2 gene, mutation, optic nerve atrophy

## Abstract

**Rationale:**

Crouzon syndrome is an autosomal dominant genetic disorder caused by mutations in fibroblast growth factor receptor 2 (FGFR2) and one of the most common types of craniosynostosis. Here we report the detection of FGFR2 mutation and its related clinical findings in 2 patients with Crouzon syndrome from a Chinese family.

**Patient concerns:**

We report a case of a 28-year-old male patient presented with the chief complaint of gradually blurring of his eyes over the last 6 months before visiting our clinics. History revealed low visual acuity in his right eye since childhood. Physical examination showed that both the patient and his mother have the appearance of craniofacial dysostosis, mandibular prognathism, ocular proptosis, short superior lip, scoliosis, and thoracic deformity.

**Diagnosis:**

Auxiliary examinations lead to the diagnosis of Crouzon syndrome with binocular optic atrophy, myelinated retina nerve fibers, and ametropia in both eyes, and amblyopia in the right eye of the male patient. The molecular genetic analysis confirmed the diagnosis by detecting a heterozygous pathogenic mutation c.1026C > G (C342W) in exon 10 of FGFR2 in both the patient and his mother, but not in any of the unaffected family members.

**Interventions and outcomes:**

None.

**Lessons:**

Our study confirms the presence of optic nerve atrophy in patients with Crouzon syndrome carrying FGFR2 C342W mutations and indicates that MRI and funduscopy should be performed to examine the optic nerve changes for patients with Crouzon syndrome.

## 1 Introduction

Crouzon syndrome is a craniofacial deformity caused by premature closure of the cranial suture.^[1]^ The incidence of Crouzon syndrome is approximately 1 in 25,000 to 60,000 live births, accounting for 4.8% of congenital craniosynostosis.^[2–4]^ About 30% to 60% of patients with Crouzon syndrome are sporadic.^[4]^ Clinically, patients with Crouzon syndrome display with poor maxillofacial formation, abnormal development of eye, and skull abnormalities due to early and premature calvaria and craniofacial bone closure, whereas their intelligence and extremities are often normal.^[5–7]^ This syndrome is a familial and autosomal dominant primary dysplasia caused by mutations in the coding region of fibroblast growth factor receptor 2 (FGFR2) at chromosome 10q25–26.^[8]^ There are 4 known fibroblast growth factor receptors (FGFRs), including FGFR1, FGFR2, FGFR3, and FGFR4, each of which consists of an extracellular domain, a transmembrane region, and a cytoplasmic tyrosine kinase domain. The FGFR2 protein consists of 3 immunoglobulin (Ig)-like subdomains (IgI, IgII, IgIII) that together regulate the signaling of FGF/FGFR, which is involved in a variety of critical biological functions, including mesoderm formation, cell growth and migration, organ development, and bone growth.^[9]^ Many mutations that occur in the FGFR2 gene are closely associated with congenital skull malformations, with mutations in exons 8 and 10 frequently observed in Crouzon syndrome being the most common.^[10]^ Here, we report the clinical assessment and FGFR2 mutational analysis in a Chinese family with autosomal dominant Crouzon syndrome.

## 2 Methods

### 2.1 Subjects and clinical evaluations

**Figure 1:**
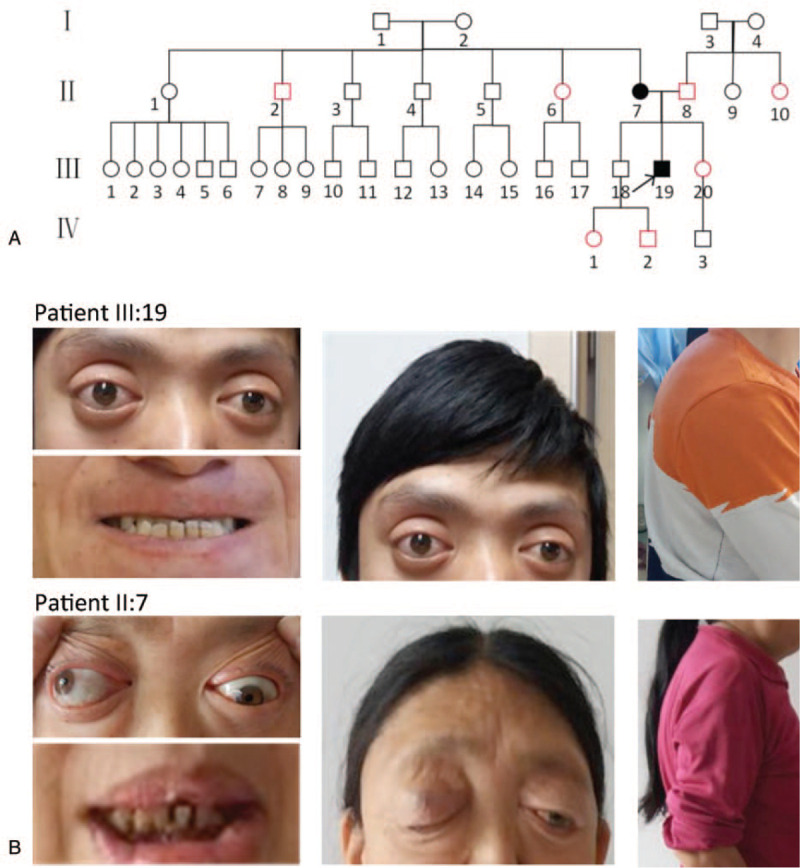
(A) The pedigree chart of the patient family with Crouzon syndrome. (B) Clinical assessment revealed that both the proband and his mother show the typical manifestation of Crouzon syndrome, including craniofacial dysostosis, ocular proptosis, hypertelorism, mandibular prognathism, short superior lip, scattered teeth, and scoliosis.

This study followed the tenets of the Declaration of Helsinki and was approved by the Ethics Committee on Human Research of the Hebei Eye Hospital. Written informed consent was obtained from the patients for publication of this case report details. One family with Crouzon syndrome consisting of 9 members across 3 generations was studied (Fig. 1). Extensive ophthalmic and physical examinations were performed on the 2 patients and related family members. Malformations such as craniofacial anomaly and poor exophthalmos occlusion were found in patients II-7 and III-19; no abnormal manifestations are seen in other family members. Visual acuity was examined using Humphrey Matrix 800 (Carl Zeiss). Anterior segment photographs were captured with TRC-NW400 Non-Mydriatic Retinal Camera (Topcon Corporation). Anterior pressure measurement was obtained by an NT-4000 tonometer (Nidek, Co. Ltd.). Optical coherence tomography (OCT) was carried out by Spectralis HRA+OCT (German Heidelberg Company). Computed tomography (CT), magnetic resonance imaging (MRI), chest-x-array, and thyroid function tests were also performed on these 2 patients.

### 2.2 FGFR2 mutations analysis

Peripheral blood leukocyte genomic DNA was isolated by phenol-chloroform extraction, dissolved in 1 × TE buffer, and stored at −20°C until used. Polymerase chain reaction (PCR) was used to amplify exons 8 and 10 of FGFR2 with the following primers: FGFR2–8 (IgIIIa) F: 5’-GGTCTCTCATTCTCCCATCCC-3’, R: 5’-CCAACAGGAAATCAAAGAACC-3’; and FGFR2–10 (IgIIIc) F: 5’-CCTCCACAATCATTCCTGTGTC-3’, R: 5’-ATAGCAGTCAACCAAGAAAAGGG-3.^[10,11]^ The polymerase and reagents used for PCR reactions were purchased from Takara Bio. The PCR reactions included 5 minutes at 95°C, followed by 40 cycles of 94°C for 45 seconds, 61°C for 45 seconds, and 72°C for 45 seconds.^[11]^ The PCR products were purified with the E.Z.N.A. Cycle Pure kit (Omega Bio-Tek) and subjected to direct sequencing on an ABI3730 sequencer (Applied Biosystems). The sequencing results were analyzed using Lasergene (DNA Star).

## 3 Case presentation

### 3.1 Clinical presentation

**Figure 2:**
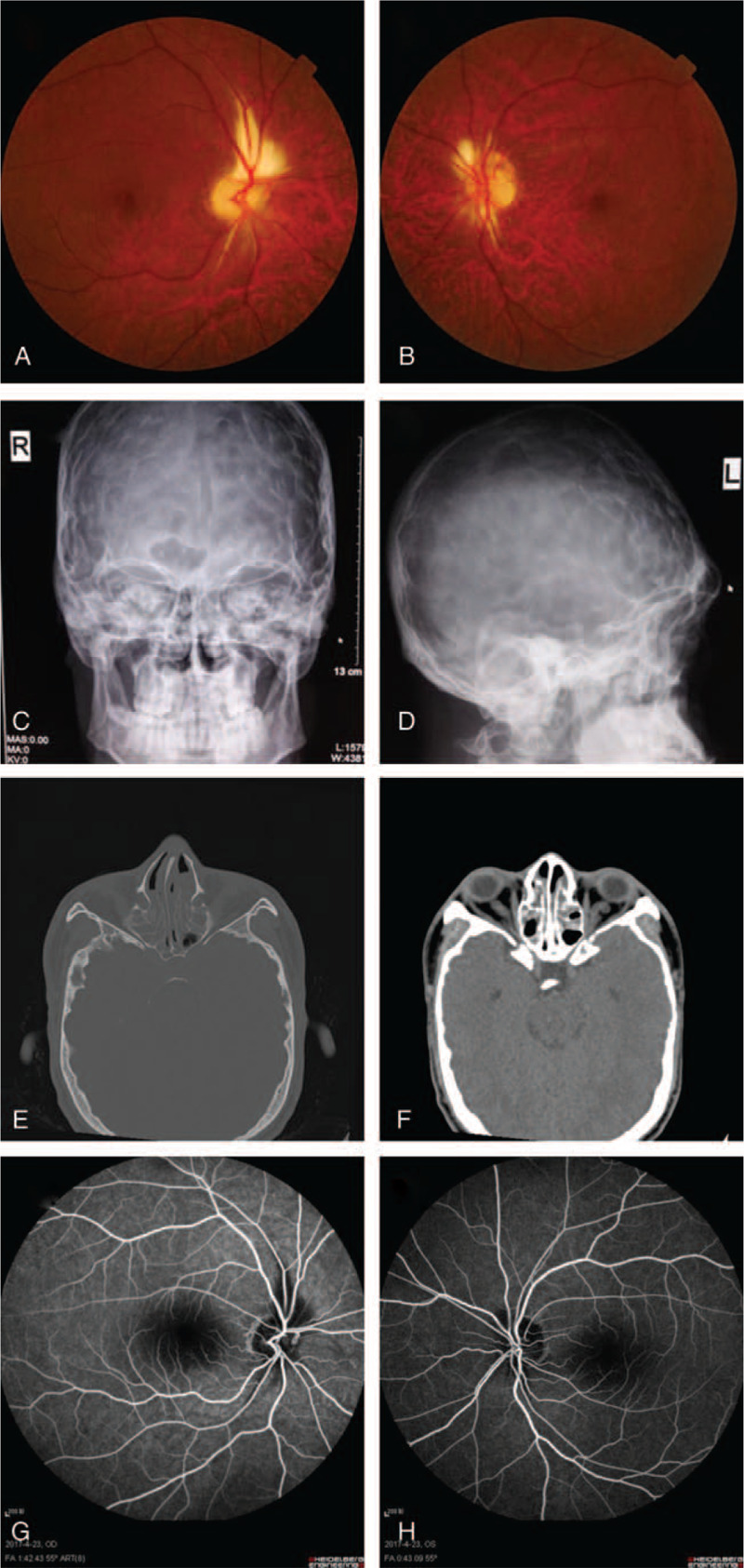
(A-B). Fundus photography of the proband's left (A) and right (B) eyes. (C-D) Cranial X-ray showed that distribution of the impressions gyrorum was seen deepening and widening in both bilateral skulls. (E-F) Orbital CT showed enlargement of medial rectus muscle in the right eye, and the optic nerve is tortuous in both eyes. Soft tissue density shadow can be seen in the intracavitary of the bilateral paranasal sinus. (G-H) FFA (fluorescein angiography) showed: binocular optic nerve atrophy, binocular retinal myelinated nerve fibers.

The proband (III-19) is a 28-year-old man who was hospitalized on April 21, 2017. The patient's self-description indicated low visual acuity in the right eye since childhood and that both of his eyes gradually became blurred over the last 6 months before visiting our clinics. Physical examination revealed that the proband had craniofacial dysostosis, mandibular prognathism, hypertelorism, ocular proptosis, short superior lip, scoliosis, thoracic deformity (Fig. 1B), and clinically normal hands and feet. The visual acuity of the proband was 0.06 (OD) and 0.2 (OS). The best-corrected visual acuity was +1.50DS = 0.05 (OD) and −1.00DS/−1.00DC × 85 = 0.4 (OS). Bilateral proptosis was observed, with no obvious limitations of ocular movement in any direction. No conjunctival congestion was observed, and the proband had clear cornea with a normal depth of atria. The pupils appeared round, about 4 mm in diameter, and showed a slow response to light, but without the lens and vitreous turbidity. Fundus photography (Fig. 2A-B) reveals that the patient's optic disc appeared pale and yellow with clear boundary; white strips of strong reflection of light could be seen in the peripapillary region. Retinal blood vessels were arranged normally. Macular tissue could be seen clearly, and it was visible of macular central fovea light reflection. The degree of exophthalmos was 25 mm (OD) and 20 mm (OS).

Cranial X-ray showed that the distribution of the impressions gyrorum was deepening and widening inside of the skull (Fig. 2C-D). Orbital CT showed enlargement of the medial rectus muscle in the right eye, and the optic nerve is tortuous in both eyes of the proband (Fig. 2E-F). Soft tissue density shadow can be seen in the intracavitary of the bilateral paranasal sinus. The defect of the visual field above both eyes and reduced photosensitivity at the lower part was observed (Supplemental Fig. 1). Craniocerebral and orbital MRI showed bilateral optical nerve distortion, consistent with the effects of bilateral optical nerve atrophy. Weak bilateral optic tracts were observed; however, no obvious abnormality in bilateral brain parenchyma was seen. Inflammation of the bilateral ethmoid sinus, maxillary sinus, and sphenoid sinus was observed. Bilateral inferior turbinate hypertrophy and deviation of nasal septum could be seen. B-mode ultrasonography demonstrated vitreous opacity in both eyes. OCT angiography of the optical nerve head blood flow revealed reduced capillary blood vessel signals around the binocular optical nerve head (Supplemental Fig. 2). The shrinkage of the retinal nerve fiber layer and thinning around the binocular optical nerve head were also observed (Supplemental Fig. 3). P-visual evoked potential (VEP) showed that the P-wave amplitude in the left eye was severely reduced. F-VEP showed that the amplitude of the P2 wave in both eyes was severely reduced, with the right eye even more severe. Fluorescein angiography revealed binocular optic nerve atrophy and myelinated retinal nerve fibers in both eyes (Fig. 2G-H). Various other test results were generally typical; no abnormalities were observed in the thyroid examination. Chest x-ray radiographs showed scoliosis and thoracic deformity, but no abnormalities in the lungs, heart, and sputum (Supplemental Fig. 4). The electrocardiogram revealed sinus arrhythmia. Based on the clinical observation, the proband was diagnosed with Crouzon syndrome with binocular optic atrophy, myelinated retina nerve fibers and ametropia in both eyes, and amblyopia in the right eye.

Physical examination showed that the proband's mother (II-7) has a similar appearance of craniofacial dysostosis, mandibular prognathism, bulging eyes, ocular proptosis, strabismus, short superior lip, and scoliosis. The visual acuity was 0.3 (OD) and 0.2 (OS). Bilateral proptosis was similarly observed, with no apparent limitations of ocular movement in any direction, and ptosis could also be seen (Fig. 1B). The conjunctiva was not congested, and the cornea was clear. The anterior chamber appeared normal. The pupils were round, approximately 3 mm in diameter. The direct response to light was slightly retarded, but without the lens and vitreous turbidity. The optic disc appeared pale with a clear boundary. The retinal blood vessels are generally healthy. The macular tissue is clear, and the central concave light reflection is visible. The proband's mother can be similarly diagnosed with Crouzon syndrome.

### 3.2 FGFR2 mutation analysis

**Figure 3:**
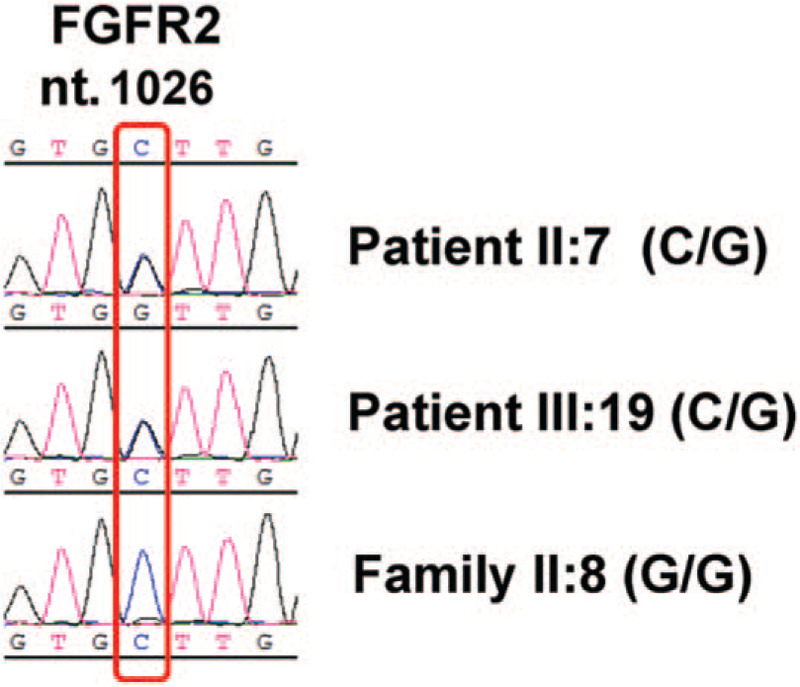
Sanger sequencing identified heterozygous missense mutation at nucleotide 1026 of FGFR2 that results in the conversion of cysteine to tryptophan (C342W).

To further confirm that this is a classic case of familial Crouzon syndrome, we took blood samples from these 2 patients and 7 other family members who do not have any symptoms. After extracting genomic DNA from peripheral blood, the 8th and 10th exons of the FGFR2 gene were amplified by PCR. The PCR products were purified and directly subjected to DNA sequencing to detect mutations. A heterozygous C-to-G transition mutation at the 1026th nucleotide of exon 10 of the FGFR2 gene was detected in the DNA of the proband and his mother, but not in any other family members without symptoms (Fig. 3). This missense mutation changes the amino acid encoded by the site from cysteine to tryptophan (C342W).

## 4 Discussion and conclusion

Crouzon syndrome is one of the most common types of craniosynostosis, and it was first reported by French neurologist Octave Crouzon in 1912.^[1]^ The main clinical features of patients with this syndrome are a premature fusion of one or more cranial sutures, and early closure of the orbit and maxillary gap, which results in the narrow cranial cavity, shallow eyelid and ocular protrusion, occipital hook, maxillary dysplasia, and mandibular relative protrusion.^[5–7]^ These anatomical changes can cause complications such as intracranial hypertension and blindness.^[12,13]^ About 95% of the mutations causing Crouzon syndrome are located in exon 8 and exon 10 of FGFR2, encoding FGFR2 IgIIIa and IgIIIc alternative splicing isoforms, respectively; in a few cases, the mutation site is found at other locations such as the FGFR3 gene.^[10]^ The causative role of FGFR2 mutations in Crouzon syndrome was first established by Reardon et al in 1994.^[14]^ In the past decades, several studies have performed molecular analysis of FGFR2 mutations in Chinese patients with Crouzon syndrome.^[11,15–25]^ Almost all of the FGFR2 mutations detected in Chinese patients with Crouzon syndrome have been previously identified in patient cohorts from Europe and the United States.^[10,25]^

In this study, FGFR2 c.1026C > G mutation was detected in the 2 patients examined, but not in any immediate family member without the observed symptoms. The c.1026C > G mutation was a missense mutation, which changed the amino acid encoded by the site from a hydrophilic cysteine to a hydrophobic tryptophan (C342W). Sequencing revealed that the mutation detected was heterozygous in the DNA of the proband and his mother. The C342W mutation was first reported by Steinberger et al in Europe and the United States in 1995.^[26]^ Li et al reported in 2016, the first case carrying this mutation in the Chinese population.^[19]^ Protein structure analysis indicated that this mutation caused changes in the extracellular Ig-III domain of FGFR2 protein, affecting the physical and biological properties of FGFR2 protein, suggesting a common pathogenic driver mutation.^[19]^ Cysteine 342 is the most frequent hotspot mutation observed in patients with Crouzon syndrome, which is often eliminated and replaced with arginine, tyrosine, serine, phenylalanine, and tryptophan.^[10]^ Mutation at this Cysteine residual may cause constitutive kinase activities, resulting in aberrant signaling, such as increased osteogenesis gene expression.^[15]^

FGFR2 mutations also occur in Pfeiffer syndrome and Apert syndrome, 2 of other typical craniosynostosis syndromes. Crouzon syndrome usually does not present limb abnormalities. The proband and his mother have clinically average hands and feet. Therefore, the diagnoses of Pfeiffer syndrome and Apert syndrome were excluded. However, in the family, the proband and his mother showed scoliosis and thoracic deformity. Scoliosis is not a typical early symptom of Crouzon syndrome but can occur later in life. The proband had more severe thoracic deformity than his mother, which was a little bit unusual.

Further, the proband displayed severe optic nerve atrophy, which is consistent with a previous report on another Chinese Crouzon syndrome patient with the same FGFR2 C342W mutation. Though it is premature to conclude that the unusual changes in optic nerves are associated with this specific mutation, an increased application of molecular genetic diagnosis might provide better insights into the genotype-phenotype correlations. Currently, the treatment of Crouzon syndrome is given based on the severity of functional and appearance-related needs, which includes acute management of symptoms and surgical interventions depending on patients’ age. Interdisciplinary and multidisciplinary care and management are required for the comprehensive treatment of patients with Crouzon syndrome to avoid repeat surgeries.

In conclusion, in this study, we present the genetic and clinical findings in 2 patients with Crouzon syndrome from a three-generation Chinese family. We confirmed the presence of optic nerve atrophy in patients with Crouzon syndrome carrying FGFR2 C342W mutations. Our study indicates that MRI, funduscopy, and OCT analysis should be performed to examine the optic nerve changes for patients with Crouzon syndrome.

## Acknowledgments

The authors also thank Dr. Huidong Shi for language editing.

## Author contributions

**Conceptualization:** Huijun Shi, Minglian Zhang

**Data curation:** Jie Yang, Qingmin Guo, Huijun Shi

**Investigation:** Huijun Shi, Jie Yang, Qingmin Guo.

**Supervision:** Huijun Shi, Minglian Zhang.

**Writing – original draft:** Qingmin Guo, Jie Yang, Huijun Shi.

**Writing – review & editing:** Qingmin Guo, Jie Yang, Huijun Shi, Minglian Zhang.

## Supplementary Material

Supplemental Digital Content

## Supplementary Material

Supplemental Digital Content

## Supplementary Material

Supplemental Digital Content

## Supplementary Material

Supplemental Digital Content
