# Supplementary material for: Clinical assessment and FGFR2 mutation analysis in a Chinese family with Crouzon syndrome: A case report
Source: Medicine (Baltimore). 2021 Mar 12;100(10):e24991. doi: 10.1097/MD.0000000000024991 (PMC7969214; doi:10.1097/MD.0000000000024991)
Supplement: Supplemental Digital Content [file medi-100-e24991-s001.pdf]

A

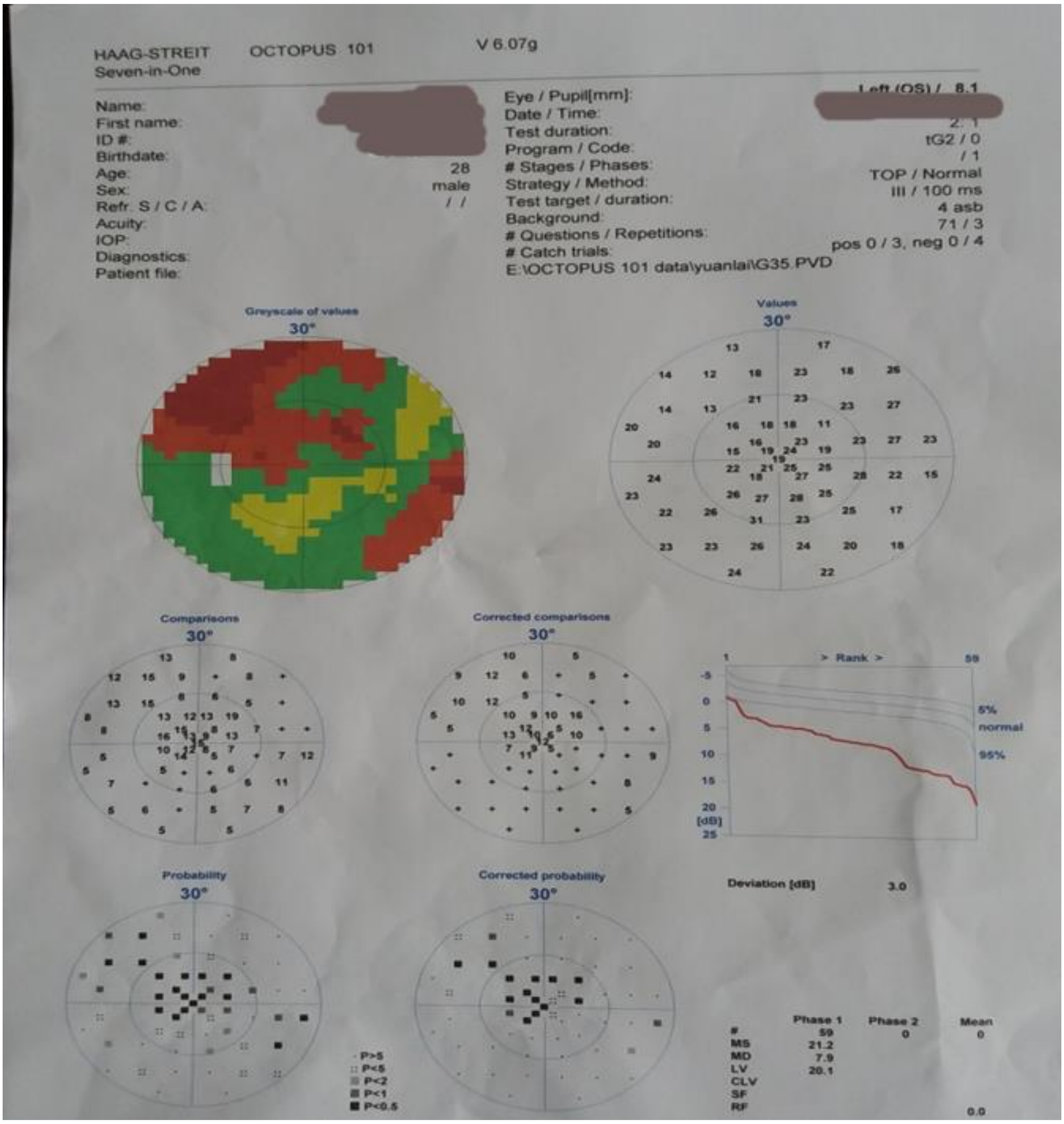
HAAG-STREIT OCTOPUS 101 V 6.07g
Seven-in-One
Name:
First name:
ID #:
Birthdate:
Age: 28
Sex: male
Refr. S / C / A: / /
Acuity:
IOP:
Diagnostics:
Patient file:
Eye / Pupil[mm]:
Date / Time:
Test duration:
Program / Code: tG2 / 0
Stages / Phases: / 1
Strategy / Method: TOP / Normal
Test target / duration: III / 100 ms
Background: 4 asb
Questions / Repetitions: 71 / 3
Catch trials: pos 0 / 3, neg 0 / 4
E:\OCTOPUS 101 data\yuanlai\G35.PVD
Greyscale of values
Values
Comparisons
Corrected comparisons
Probability
Corrected probability
30°
Rank
5%
normal
95%
[dB]
Deviation [dB] 3.0
Phase 1
Phase 2
Mean
MS 21.2
MD 7.9
LV 20.1
CLV
SF
RF 0.0
P>5
P<5
P<2
P<1
P<0.5

B

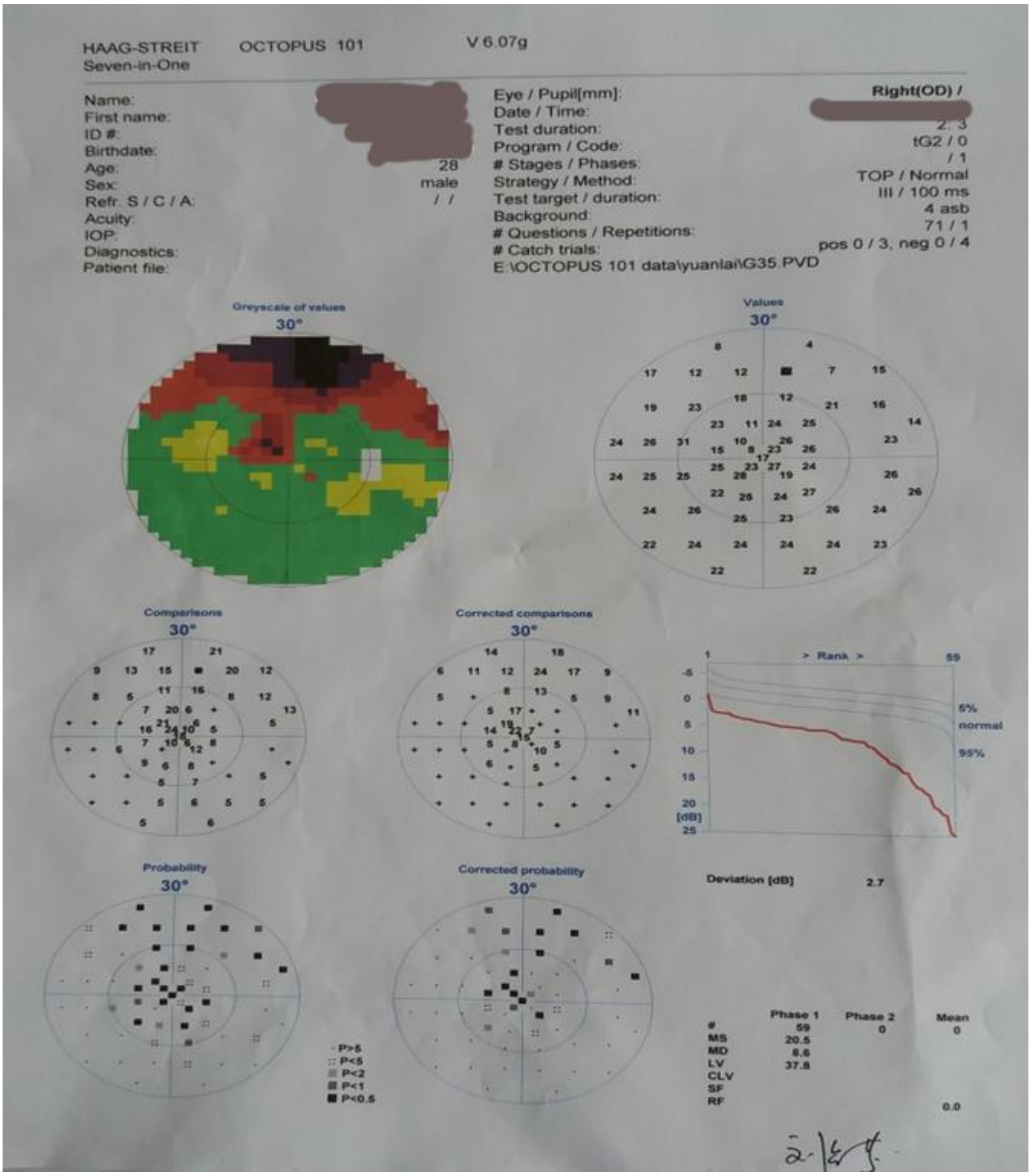

Supplemental Figure 1： The visual field analysis of proband's left (**A**) and right (**B**) eyes by OCTOPUS 101 Perimeter Visual Field Analyzer showed defects of the visual field above both eyes and reduced photosensitivity at the lower part.
