# Supplementary material for: Clinical assessment and FGFR2 mutation analysis in a Chinese family with Crouzon syndrome: A case report
Source: Medicine (Baltimore). 2021 Mar 12;100(10):e24991. doi: 10.1097/MD.0000000000024991 (PMC7969214; doi:10.1097/MD.0000000000024991)
Supplement: Supplemental Digital Content [file medi-100-e24991-s002.pdf]

A

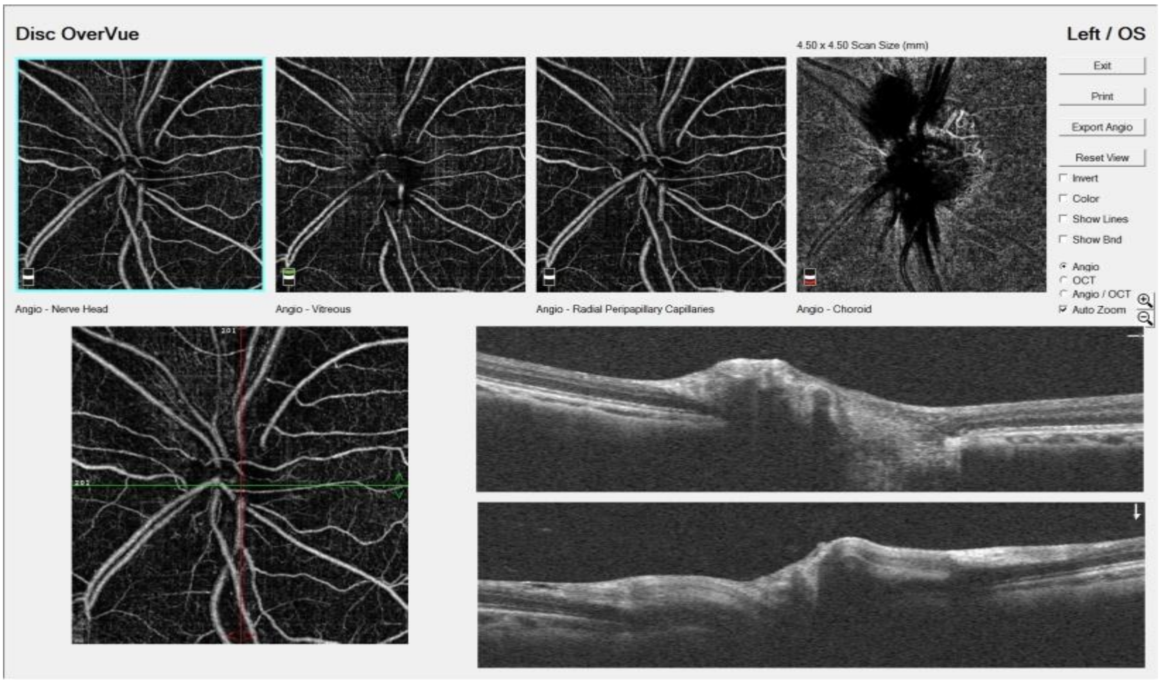

B

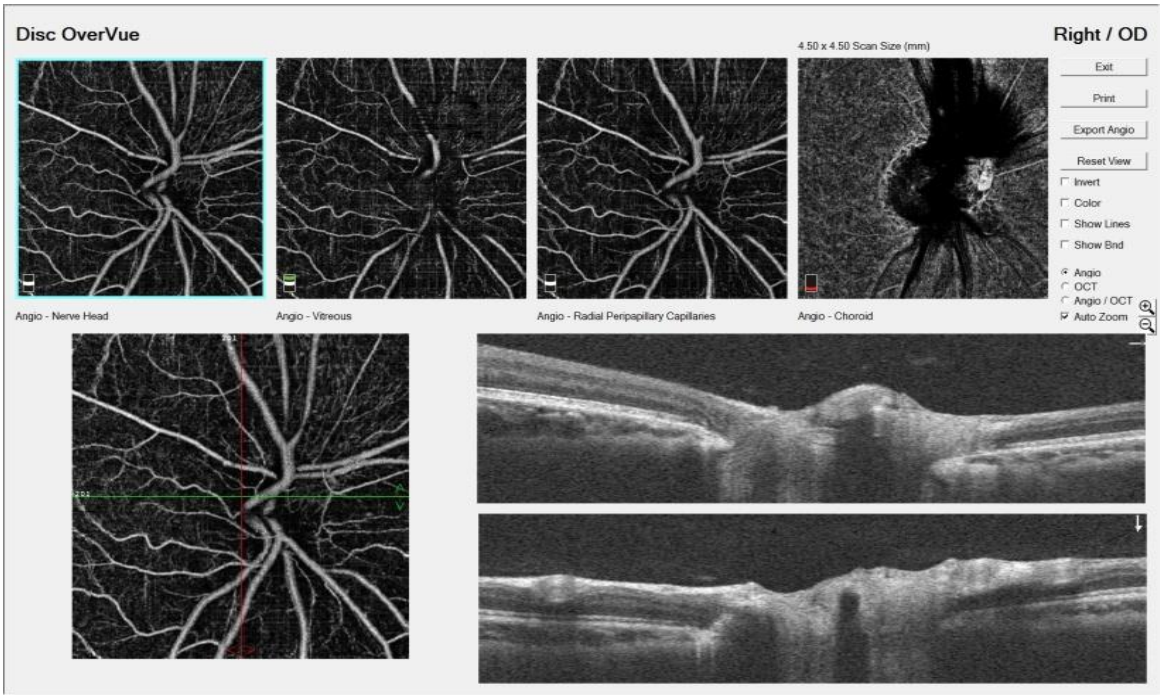

Supplemental Figure 2： OCT angiography of the optic disc blood flow in left (**A**) and right (**B**) eyes. Decreased capillary signal around the optic disc in both eyes. Reduced capillary blood vessel signals around the binocular optical nerve head were observed.
