# Supplementary material for: Clinical assessment and FGFR2 mutation analysis in a Chinese family with Crouzon syndrome: A case report
Source: Medicine (Baltimore). 2021 Mar 12;100(10):e24991. doi: 10.1097/MD.0000000000024991 (PMC7969214; doi:10.1097/MD.0000000000024991)
Supplement: Supplemental Digital Content [file medi-100-e24991-s003.pdf]

A

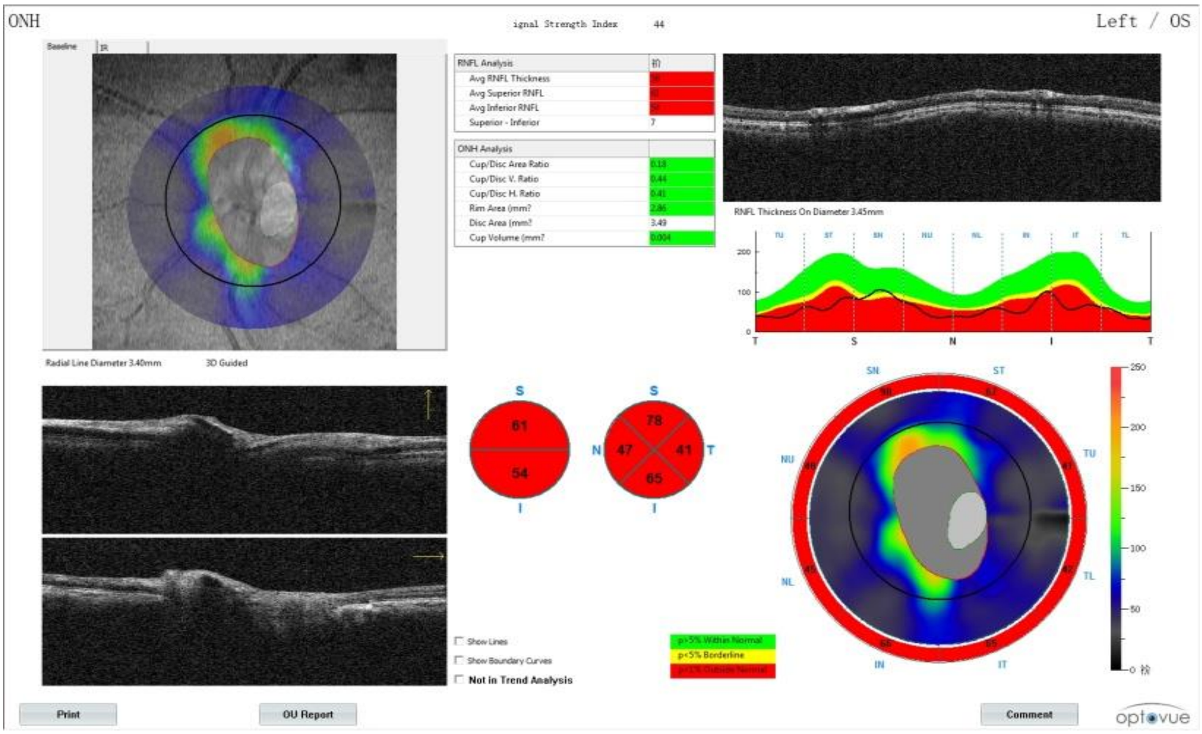

B

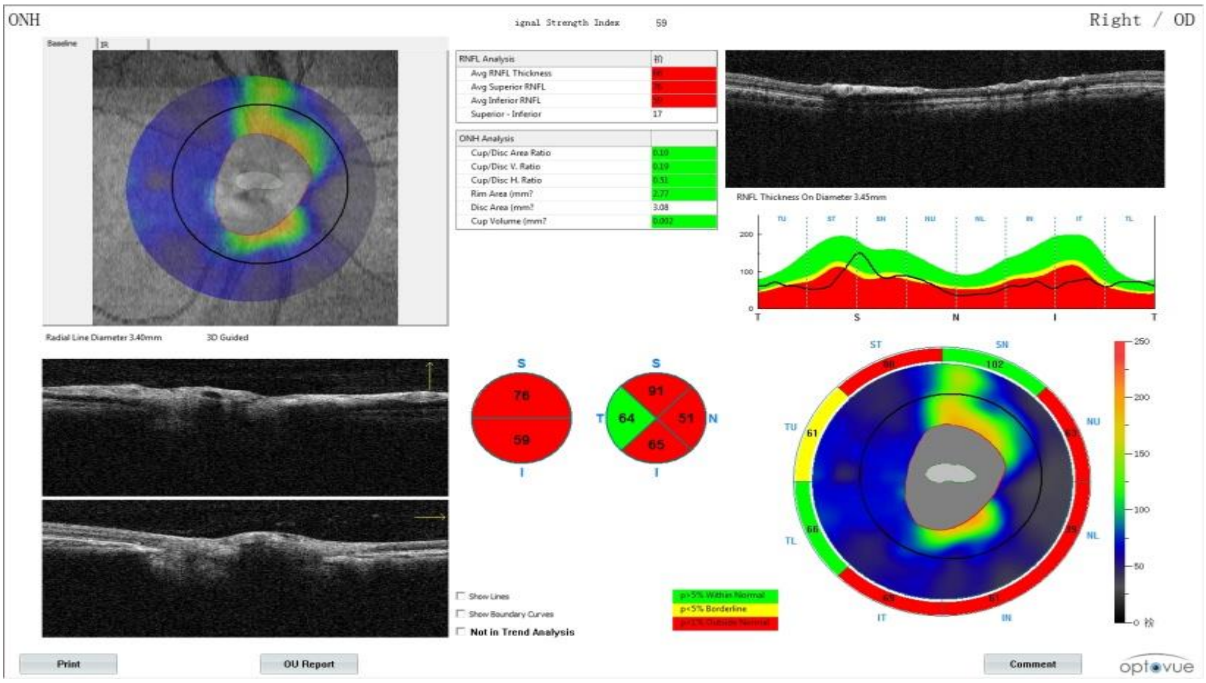

Supplemental Figure 3: RNFL analysis of the left (**A**) and right (**B**) eyes revealed shrinkage of the RNFL and thinning around the binocular optical nerve head.
