# Supplementary material for: Clinical assessment and FGFR2 mutation analysis in a Chinese family with Crouzon syndrome: A case report
Source: Medicine (Baltimore). 2021 Mar 12;100(10):e24991. doi: 10.1097/MD.0000000000024991 (PMC7969214; doi:10.1097/MD.0000000000024991)
Supplement: Supplemental Digital Content [file medi-100-e24991-s004.pdf]

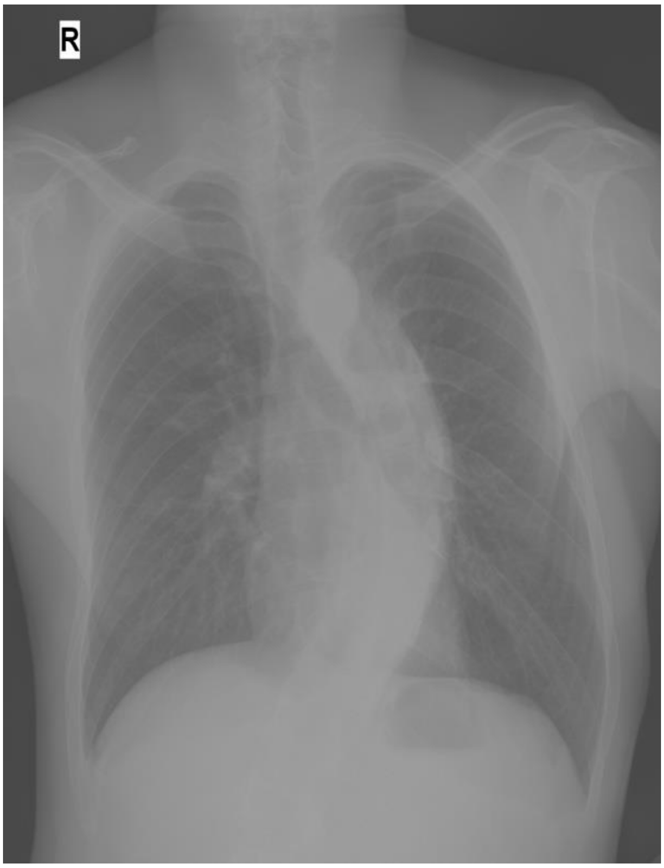

Supplemental Figure 4: Chest radiographs of the proband showed the signature of scoliosis and thoracic deformity. However, there were no abnormalities in both lungs, heart, and palate.
